# Ecological risk and enrichment of potentially toxic elements in the soil and eroded sediment in an organic vineyard (Tokaj Nagy Hill, Hungary)

**DOI:** 10.1007/s10653-021-01076-w

**Published:** 2021-09-03

**Authors:** Nhung Thi Ha Pham, Izabella Babcsányi, Andrea Farsang

**Affiliations:** 1 Department of Geoinformatics, Physical and Environmental Geography, University of Szeged, Egyetem u. 2-6, Szeged, 6722 Hungary; 2 Faculty of Environmental Sciences, University of Science, Vietnam National University, Hanoi, 334 Nguyen Trai Street, Thanh Xuan District, Hanoi, Vietnam

**Keywords:** Bioavailability, Ecological risk assessment, Potentially toxic element, Vineyard soil, Sediment, Soil erosion

## Abstract

Potentially toxic elements (PTEs), such as Cu, Zn, Pb, Ni, Cr, and Co, can accumulate in vineyard soils due to repeated uses of inorganic pesticides and chemical or organic fertilizers. In sloping vineyards, PTEs can also be moved by soil erosion resulting in their accumulation in low-energy zones within the landscape, adversely affecting the soil environment. Our study evaluated the ecological risk related to the pseudo-total and bioavailable PTE contents (Zn, Pb, Co, Ni, Cr, and Cu) in the soil and eroded sediment samples from an organic vineyard in Tokaj (NE Hungary). The contamination status and the ecological risk of target PTEs were assessed by calculating the contamination factor, the pollution load index, the ecological risk factor, and the ecological risk index. The median pollution load indices of 1.15, 1.81, and 1.10 for the topsoil, the sediments, and the subsoil, respectively, demonstrate a moderate multi-element contamination case in the organic vineyard. Target PTEs tented to show increased concentrations in eroded sediments with the highest enrichment ratio (3.36) observed for Cu (Cu in the sediment/Cu in the topsoil), revealing a preferential movement of Cu-rich soil particles by overland flow. Moreover, PTEs were present in the sediments in more bioavailable forms (except Ni, Cr), assessed by an extraction procedure with EDTA. The ecological risk index (< 90) based on the studied PTEs showed an overall low ecological risk in the vineyard. Copper was the predominant factor of the ecological risk. Moreover, the highest ecological risk factor (24.6) observed for the bioavailable Cu content in an eroded sediment sample (representing 82% of the total ecological risk) shows that Cu accumulation in sloping vineyards is an ecological risk, particularly in the sedimentation zones. The high proportions of bioavailable Cu in the vineyard’s soil represent an increasing ecological risk over time, related to repeated treatments of vine plants with Cu-based pesticides.

## Introduction

Agricultural production is influenced by natural drivers such as soil, topography, climate, and landscape (Li & Zhou, 2015; Schaller et al., 2018). These factors play a key role in cultivation culture and product quality, especially in sustainable agriculture (Schaller et al., 2018). In addition, the “terroir,” which is the combined effect of several local factors, including the soil, has a substantial role in determining the grape quality and wine sensory properties (Czigány et al., 2020; Ferrretti, 2019; Qi et al., 2019). However, agricultural activities are a primary source of potentially toxic elements (PTEs) in cultivated soils, generating permanent pollution of large areas (Chen et al., 2015; Li et al., 2015). Particularly, in the vineyards, soil pollution with PTEs from the continuous use of cupric fungicides and fertilizers is a predominant issue (Preston et al., 2016). With the long-term use of chemical fertilizers, fungicides, and organic byproducts, viticulture can exert a considerable risk of soil contamination that may ultimately impact product quality (Mirzaei et al., 2019; Preston et al., 2016). Fertilizers supply non-negligible quantities of PTEs, such as Cu, Zn, and Ni, that are essential micronutrients for plant growth. Yet, their increased contents in the cultivated soils may reach toxicity thresholds, exceeding the optimal range for plants and soil microorganisms (Dhaliwal et al., 2019). The intensive use of fertilizers and copper-based fungicides have already induced superabundance of PTEs (such as Cu, Zn, Cd, Pb, Cr, Ni, Hg, and As) in many vineyard soils (Chen et al., 2015; Liang et al., 2015; Mirzaei et al., 2019). Specific accumulation patterns and bioavailability of PTEs have been assessed in previous studies for evaluating the contamination level, the environmental pollution risk, and the toxicity of PTEs to crops (Borgese et al., 2013; Liu et al., 2005; Nunes et al., 2014; Sipos 2004; Violante et al., 2010). However, organic vineyards have been rarely targeted in those studies. Therefore, further research on PTEs and the associated ecological risk is necessary to supply information on the sustainability of current organic farming practices in vineyards.

Erosion has been indicated as one of the major threats that affect agricultural soils (Biddoccu et al., 2016). In particular, hilly vineyards are easily subject to soil erosion, depending on the soil management system, and have the highest measured soil losses compared to rainfed cereals, olives, eucalyptus plantation, or scrubland (Biddoccu et al., 2016; Kosmas et al., 1997; Novara et al., 2011). Tillage or weed control with herbicides usually used in vineyards leave the bare soil exposed to rainfall and runoff during all or part of the year (Biddoccu et al., 2020). As a result, in the sloping vineyard, soil erosion and cultural practices drive the spatial distribution of macro-and micronutrients in the soil (Chevigny et al., 2014; Czigány et al., 2020). The spatial distribution of the fertilizer- and pesticide-derived PTEs in vineyard soils is also affected (Manaljav et al., 2021). Soil erosion and redeposition of PTE-rich sediment particles increase the risk of contamination of non-target environments (He et al., 2004; Ribolzi et al., 2002). Indeed, vineyard soils undergo wash-off during intense rainfall events, especially in steep areas, which may raise environmental and ecological concerns locally (in the soil) and off-site when reaching vulnerable aquatic environments (Martínez-Casasnovas & Ramos, 2006; Koulouri & Giourga, 2007). Hence, the impact of soil erosion on the distribution and transport of PTEs should be carefully evaluated based on assessing both the total and the bioavailable PTE contents in the soil and eroded sediments in vineyards.

In recent years, ecological risk concerns associated with the accumulation of PTEs have arisen and become an environmental burden (Arfaeinia et al., 2019; Milićević et al., 2018; Mirzaei et al., 2019). The ecological risk of PTEs calculated based on contamination factors and biological toxicity factors has been widely used for soil and sediment contamination investigations (Canuto et al., 2013; Hakanson, 1980). Also, for a better understanding of the PTE-related risks, their bioavailable concentrations should be considered. Therefore, in our study, pollution load and ecological risk indices are calculated based on the pseudo-total and the bioavailable contents of PTEs. In vineyards subject to erosion, PTEs can be significantly relocated and accumulated in low-energy zones of the landscape. Hence, the enrichment and ecological risks of PTEs should also be examined in eroded sediments. In some previous works (Liang et al., 2015; Milićević et al., 2018; Mirlean et al., 2007; Mirzaei et al., 2019; Romíc et al., 2004), the PTEs selected in our work (Zn, Pb, Co, Ni, Cr, and Cu) were indicated as potential soil pollutants with ecological risk concerns. Thus, we aimed to assess the pseudo-total and bioavailable fractions of Zn, Pb, Co, Ni, Cr, and Cu in the soil and eroded sediments collected in an organic vineyard on the hillslope of the Tokaj Nagy Hill, a part of the Tokaj-Hegyalja Wine Region (NE Hungary). The specific objectives of the study are (1) to evaluate relationships between soil properties (pH, soil organic matter (SOM), carbonate content, and soil texture) and the pseudo-total and bioavailable contents of target PTEs, (2) to determine the contamination status and enrichment of selected PTEs in the soil and eroded sediments, and (3) to assess the ecological risk of target PTEs.

## Material and methods

### Study area and sample collection

The study area is part of the Hétszőlő vineyard situated at the south-facing hillslopes of Tokaj Nagy Hill (NE Hungary). Tokaj Nagy Hill is one of the easternmost members of the inner Carpathian volcanic range in Hungary (Lóczy, 2015; Molnár et al., 2010; Zelenka et al., 2004; Szepesi et al., 2018). The primary parent material is a late Miocene stratovolcano, built up of pyroxene dacite lava flows and subordinate pyroxene dacite tuffs (Harangi & Lenkey, 2007; Novák et al., 2014; Zelenka et al., 2004). Loess deposits cover volcanic rocks of the Tokaj Nagy Hill with their thickness ranging from a few centimeters to 20 m (Kerényi, 1994; Lóczy, 2015; Novák et al., 2014). An open-pit quarry (dacite stone quarry) abandoned in the 1980s is situated at ~ 500 m from the studied site. The climate is humid continental, characterized by cold winters and warm summers. The mean annual precipitation varies between 590 and 610 mm, with a clear maximum value (360 mm falls) in the summer months (Dövényi et al., 2010). The mean and the maximum rainfall intensity during the year of study (2019) were 1.3 mm/h and 27.8 mm/h. The mean annual temperature is 8.5 °C at the summit and reaches almost 10 °C at the base of the Tokaj Nagy Hill (Novák et al., 2014).

**Fig. 1:**
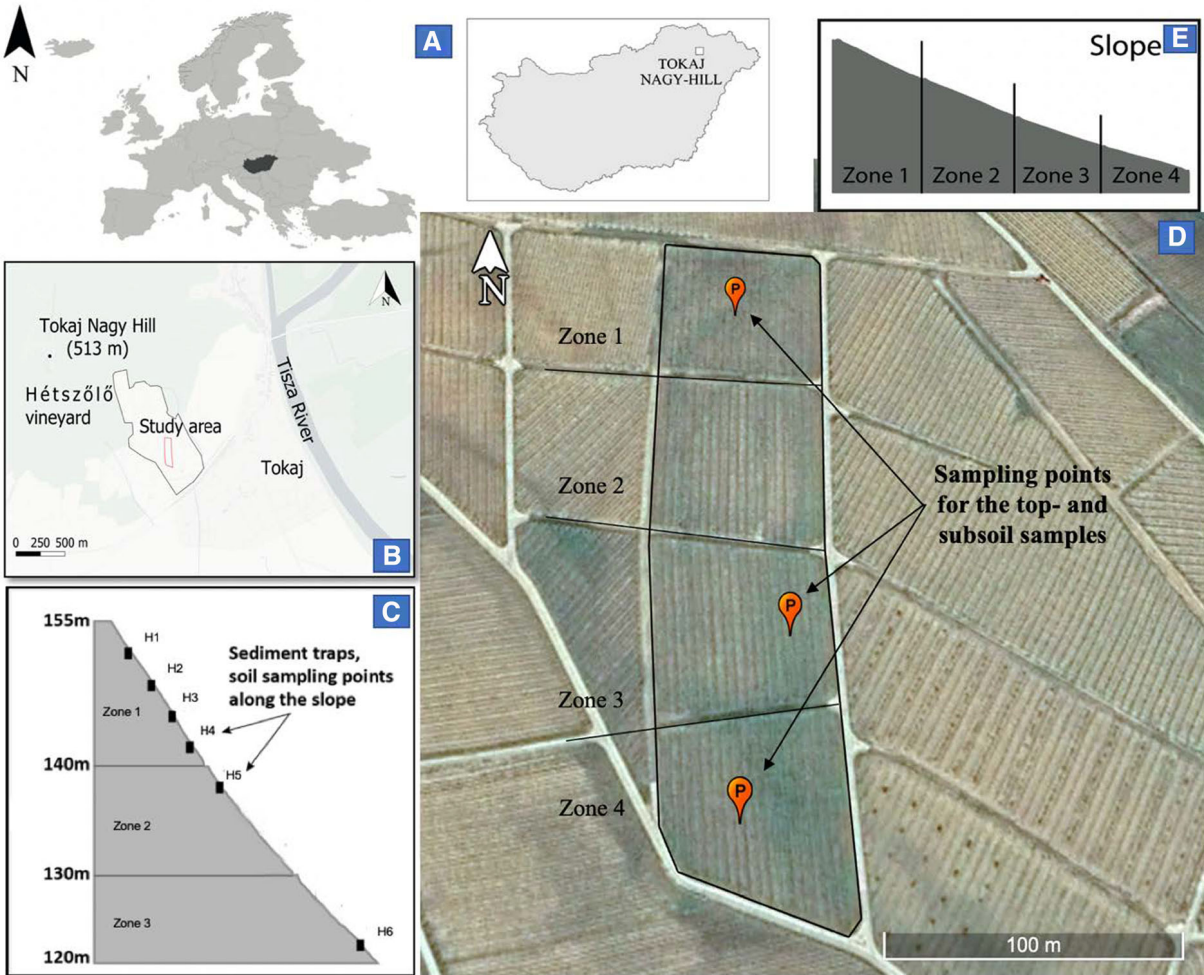
**A** Location of the study vineyard, situated in the winegrowing region of Tokaj-Hegyalja (NE Hungary); **B** The intermediate map for Tokaj; **C** and **E** The slope profiles and the position of sediment traps and soil sampling along the main slope; **D** Sampling points for the top- and subsoil

The study site (Fig. 1) is a 1.8 ha vineyard plot with a mean slope of 8° and a hillslope length of 270 m. A mixture of grass and herbaceous plants cover the vine inter-rows for limiting soil erosion. The soil is tilled (0–20 cm) biannually in the inter-rows; meanwhile, tillage is done twice a year underneath the vine rows. Mowing is practiced to control weed, and no herbicides are used. The vineyard is managed according to the guidelines of organic farming. For years, fresh cattle manure was used at a dose of 0.3 t/ha every 3–4 years for soil fertilization. For the last ten years, the local vinegrowers have applied cattle manure pellets. Treatments with Cu-based fungicides are regularly done in a typical 4 kg/ha/year dose (metallic Cu). We suppose that most of the Cu-pesticide applications have taken place over the past 28 years since replanting the vineyard in 1993. The soil in the vineyard is a Calcaric Regosol (Siltic, Ochric) according to the World Reference Base for Soil Resources (2014). The soil in the vineyard is weakly developed due to the intense soil erosion that prevents the formation of apparent diagnostic horizons. The soil-forming parent material is loess.

In March 2019, the topsoil (< 20 cm layers: 0–10 cm and 10–20 cm) and subsoil (> 20 cm layers: 20–30 cm; 30–40 cm; 60–80 cm; 120–140 cm; and 180–200 cm) samples (21 soil samples) were collected at three locations in the vineyard using a hand auger (Fig. 1). Subsoil samples collected from boreholes at 180–200 cm depth are considered as the parent material devoid of impacts from plant protection treatments and not reaching the dacite base rock. At the same time, six sediment traps were deployed along the main slope. Additionally, a composite topsoil sample from 0–10 cm soil layer was collected from the local forested site as a reference soil (not being impacted by vine-growing). In May 2019, eroded sediment samples from the traps were collected along with six topsoil samples (0–20 cm) near each trap (Fig. 1). During the sample collection period (one week), the average precipitation depth was 2.58 mm per rainfall event; the mean rainfall intensity was 1.55 mm/h, and the maximum intensity was 5.5 mm/h. The total rainfall depth was 15.5 mm. Rainfall data are continuously recorded on-site within the area of the Hétszőlő vineyard using a rain gauge set up (Type BCU LITE2, Boeras Ltd., Hungary).

### Sample treatment and analyses

The collected soil samples were air-dried, while the eroded sediment samples were oven-dried at 80 °C. All samples were disaggregated in a mortar with a pestle and then sieved to pass through a 2-mm sieve. The hygroscopic moisture of each sample was determined at 105 °C in the oven for 24 h (overnight). The soil pH (d.w.) was measured in a mixture (1:2.5) of soil–deionized water using a digital pH meter (Inolab pH 720) (MSZ-08-0206-2, 1978) (± 0.05). The SOM content was analyzed by a UV–VIS spectrophotometer (a type Spectronic Helios-γ, Thermo Fisher Scientific), following H_2_SO_4_ -aided oxidation of the organic matter with 0.33 M K_2_Cr_2_O_7_ (MSZ-21470–52, 1983) (± 2%). The carbonate content (in percentage of dry matter weight, ± 8%) was determined using a calcimeter according to the Scheibler method using 10% HCl solution for the reaction (Bojar et al., 2020). The values of the plasticity index according to Arany (Arany Plasticity Index) are used to identify the soil texture following the Hungarian Standard-MSZ-08-0205, 1978. The method consists of determining the amount (cm^3^) of deionized water added to 100 g of air-dry soil sample until reaching the upper limit of its plasticity. The particle-size distribution was determined by the pipette method following treatment with 0.1 M sodium pyrophosphate (MSZ-08–0205, 1978).

For the pseudo-total PTE analysis (Zn, Pb, Co, Ni, Cr, and Cu), samples were finely ground in an agate ball mill (for soil samples) or in a mortar with a pestle (for sediment samples) to pass through a 250 µm sieve. Then, 0.5 g sample material was weighed into a PFA vessel and 7 ml aqua regia (HNO_3_/HCl = 1:3 = 1.75 ml:5.25 ml) was added. Clean acids were applied (Normatom® for trace metal analysis, VWR Chemicals) for digestion and standard dilution. Samples were digested in a microwave oven (Anton Paar Multiwave 3000) as described elsewhere (Szolnoki & Farsang, 2013). Concentrations of PTE in digested samples were determined by an inductively coupled plasma optical emission spectrometer (ICP-OES) (Optima 7000 DV, PerkinElmer) (with ± 10% uncertainty), using yttrium as an internal standard.

The bioavailable fraction of PTEs (Zn, Pb, Co, Ni, Cr, and Cu) was quantified using an extraction procedure with a strong chelating agent (0.05 M Na_2_-EDTA) (Carter & Gregorich, 2007). All centrifuge tubes were acid-washed and rinsed with purified 0.05 M Na_2_-EDTA followed by a complete ultrapure water rinse. For the extraction procedure, 1.0 g sample material was weighed in a 50 ml centrifuge tube and 25 ml of purified 0.05 M Na_2_-EDTA was added. Samples were rotated in an end-over-end shaker (Stuart SP3 Rotator) (at 15 rpm for 1 h), centrifuged (at 2500 g for 20 min), filtered (at 0.45 μm), and then kept at 4 °C until ICP analysis (as described by Carter & Gregorich, 2007). Before determining bioavailable PTE concentrations by ICP-OES, all samples were diluted 20-times with ultrapure water. Calibration standards were prepared in the same matrix as the diluted extract solution.

### Soil contamination and risk assessment indices

#### Contamination factor (Cf)

Assessing the degree of contamination by a given PTE is performed by comparing the pollutant element concentration with unpolluted reference material as background concentration (Antoniadis et al., 2017; Mirzaei et al., 2019; Rinklebe et al., 2019). Thence, in our study, Cf was calculated by dividing the content of an individual PTE in the vineyard soil/sediment by its background concentration in the local forest as the reference soil. The equation is as follows:

1 $$\mathrm{Cf }= \frac{{\mathrm{C}}_{\mathrm{s}}}{{\mathrm{C}}_{\mathrm{RefS}}}$$

(Antoniadis et al., 2017a, b, 2019; Rinklebe et al., 2019). where *C*_s_ is an element content (mg/kg); C_RefS_ is the reference concentration of the element in the local forest soil. The classification of the contamination factor is described in 4 levels (Hakanson, 1980; Islam et al., 2015): low contamination (Cf < 1); moderate contamination (1 ≤ Cf < 3); considerable contamination (3 ≤ Cf < 6); high contamination (6 ≤ Cf).

#### Pollution load index (PLI)

The PLI was applied here for evaluating the overall pollution degree of soils and sediments considering all target PTEs. PLI (unitless) was calculated by multiplying the Cf of each element (Antoniadis et al., 2019; Mirzaei et al., 2019) following Eq. 2:

2 $$\mathrm{PLI }=\sqrt[\mathrm{n}]{{\mathrm{Cf}}_{1} \times {\mathrm{Cf}}_{2} \times {\mathrm{Cf}}_{3} \times \dots \times {\mathrm{Cf}}_{\mathrm{n}}}$$

where Cf_1_ to Cf_n_ are the contamination factors of the first to the n^th^ PTE in the vineyard soil/sediment. The degree of contamination based on the PLI is described in 5 levels (Mirzaei et al., 2019): non-pollution (PLI = 0); non to moderate pollution (0 < PLI ≤ 1); moderate pollution (1 < PLI ≤ 2); moderate to high pollution (2 < PLI ≤ 3); high pollution (3 < PLI).

#### Ecological risk factor (Ei)

Hakanson (1980) first used the ecological risk factor to evaluate the ecological risk of pollutants in sediments. Thereafter, authors have widely included these indices in their studies investigating the environmental consequences of soil contamination (Arfaeinia et al., 2019; Islam et al., 2015; Mirzaei et al., 2019; Sun et al., 2010). The ecological risk factor (Ei) is applied to assess the ecological risk of an individual metal according to the Hakanson ecological risk index, as follows:

3 $$\mathrm{Ei}= {\mathrm{Cf}}_{\mathrm{i}} \times {\mathrm{T}}_{\mathrm{i}}$$

where Cf_i_ is the single PTE contamination factor (calculated by Eq. 1) and T_i_ is the biological toxicity factor of an individual target PTE. Based on the recommendations in Luo et al., 2007, standard response coefficients (T_i_) for the toxicity of the target PTEs are as follows: Zn = 1, Pb = Co = Cu = 5, Ni = 6, and Cr = 2. These values were also used in our research.

The ecological risk index (ERI) is estimated according to Eq. 4 to evaluate the overall ecological risk of all studied PTEs (Islam et al., 2015; Mirzaei et al., 2019).

4 $${\text{ERI}} = \sum\nolimits_{{i = 1}}^{6} {Ei\,(i = 1 - 6)}$$

where Ei is the ecological risk factor for each individual PTE involved in the study.

The ecological risk is grouped into 5 categories for the Eis and 4 for the ERI values (Chen et al., 2015; Islam et al., 2015; Liu et al., 2014):

Ei < 40: Low ecological risk; 40 ≤ Ei < 80: Moderate ecological risk; 80 ≤ Ei < 160: Considerable ecological risk; 160 ≤ Ei < 320: High ecological risk; 320 < Ei: Very high ecological risk.

ERI < 90: Low risk; 90 ≤ ERI < 190: Moderate risk; 190 ≤ ERI < 380: Considerable risk; 380 ≤ ERI: High risk.

#### Enrichment ratio (ER)

To compare the concentrations of the studied PTEs in the eroded sediments with those in the topsoils (< 20 cm depth), enrichment ratios (ER = element content in the sediment over that in the topsoil) were used (Farsang et al., 2012; Farsang & Barta, 2004; Fernández-Calviño et al., 2012).

### Statistical analysis

The relationships between the top- and subsoil characteristics and the PTE concentration data were explored using the Spearman rank correlation test. The significance level was set at *p* < 0.05. One-way analysis of variance (ANOVA) was used to test differences among means in the top- and subsoil parameters. Descriptive statistical analysis of the target parameters was performed using IBM SPSS software (SPSS Inc., 2009).

## Results and discussion

### Soil properties

The statistics of measured parameters of the vineyard topsoil (0–20 cm) and subsoil (> 20 cm), such as pH, soil texture, carbonate, and soil organic matter (SOM) contents, are presented in Table 1. The soil parameters did not vary significantly between the top- and subsoil layers, except for the SOM content. The topsoils in the studied vineyard are characterized by a poor SOM content (1.75 ± 0.51% in the topsoil), further decreasing downwards in the profile (0.99 ± 0.40% in the subsoil). The soil was moderately alkaline (pH ranging from 7.92 to 8.27) owing to its carbonate content that varies from 3.4 to 6.2%. The Arany Plasticity Index showed that the soil texture was a sandy loam without any marked difference between the topsoil and the subsoil. According to the particle-size distribution, both the top- and subsoil have silty loam textural characteristics with slightly lower clay content in the topsoil (Table 1). Soil erosion probably resulted in losses of the fine soil particles from the soil surface, hence the clay content got slightly depleted in the topsoil over time (Biswas et al., 2018; Udom et al., 2018).

**Table 1:** Descriptive statistics of soil parameters in the studied vineyard top- (0–20 cm depth) and subsoil (> 20 cm depth)

	SOM (%)	pH	CaCO_3_ (%)	Arany Plasticity Index	Clay %	Silt %	Sand %
*Topsoil (N* = *12)*							
Mean	1.75	8.10	4.3	34	17	69	14
Median	1.88	8.12	4.3	34	16	70	13
Min	0.72	7.92	3.4	32	11	58	6
Max	2.37	8.27	5.5	37	20	75	26
SD	0.51	0.13	0.7	1	3	5	6
*Subsoil (N* = *15)*							
Mean	0.99	8.17	4.5	34	19	68	13
Median	0.96	8.14	4.6	34	19	68	13
Min	0.37	7.85	3.3	32	14	60	8
Max	1.52	8.52	6.2	36	26	75	25
SD	0.40	0.19	0.8	1	4	4	5

### Potentially toxic elements in the studied vineyard soil and eroded sediments

**Fig. 2:**
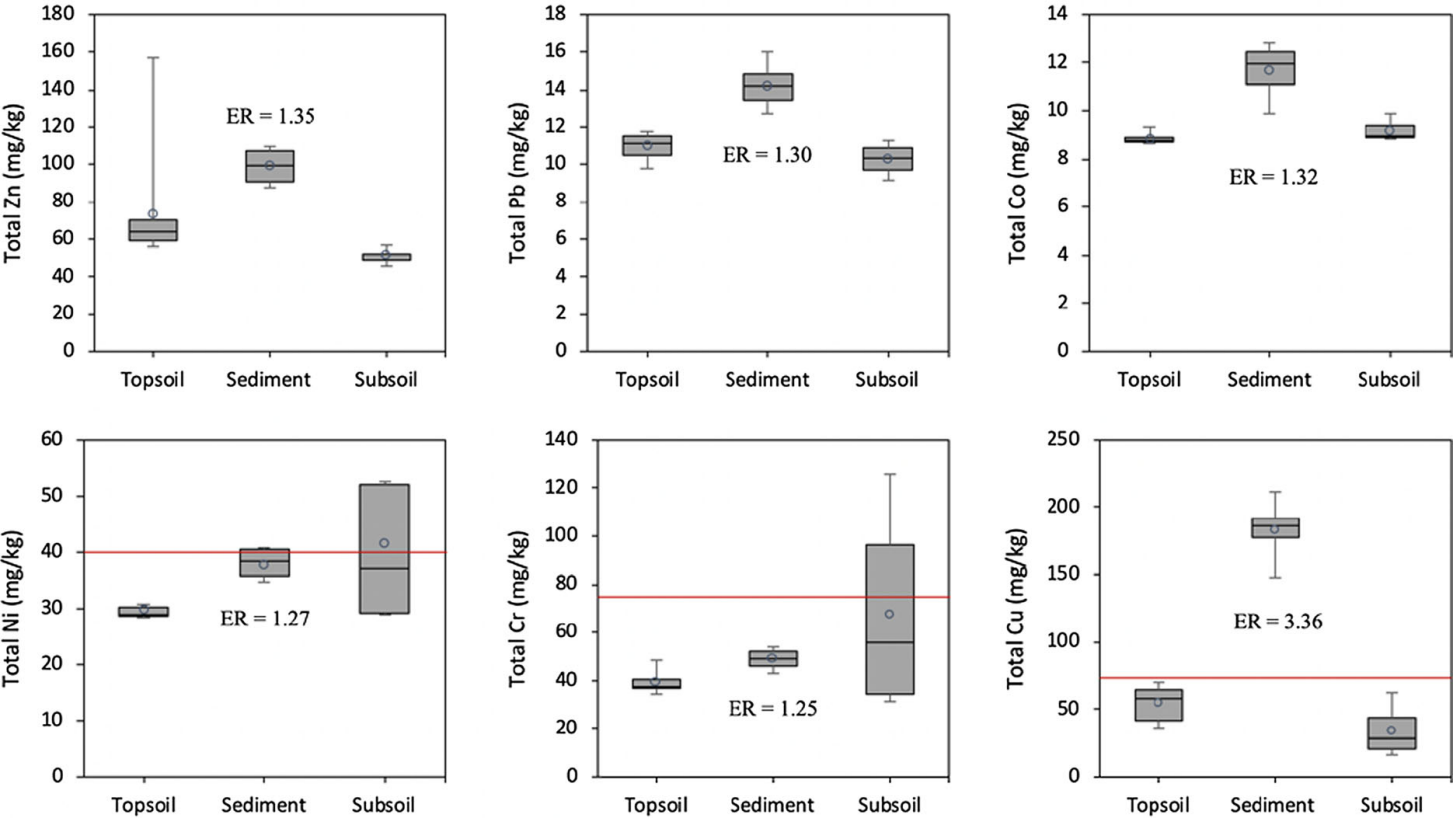
Pseudo-total PTEs contents (mg/kg) in the top- (0–20 cm) and subsoil (> 20 cm) and the sediment in the vineyard in Tokaj. ER stands for the average of enrichment ratios (ER) in sediments compared to the topsoil. The red line represents the pollution limit values (B) for soils and sediments according to Hungarian standards

The soil-bound PTE contents in the vineyard top- and subsoil are summarized in Fig. 2. Topsoil median contents of Zn (64.3 in the top- and 51.8 mg/kg in the subsoil) and Cu (58.5 in the top- and 28.3 mg/kg in the subsoil) were higher than their median subsoil contents. However, Ni (29.1 in the top- and 37.7 mg/kg in the subsoil) and Cr (37.0 in the top- and 55.6 mg/kg in the subsoil) showed opposite trends. The pseudo-total contents of Pb (11.1 in the top- and 10.3 mg/kg in the subsoil) and Co (8.8 in the top- and 9.0 mg/kg in the subsoil) did not differ significantly. The higher concentration of Zn and Cu in the topsoil indicated their enrichment in the vineyard soil. Treatments with Zn-containing fertilizers are regularly done in the vineyard, spreading an annual Zn dose of approximately 30 g/ha. Alkaline soils are often devoid of adequate Zn levels (Fernández-Calviño et al., 2012); hence, foliar fertilizers are commonly used for preventing Zn deficiency in crops. We further compared the measured levels with the pollution limit value (B) for soils and sediments figuring in Hungarian standards (Joint Decree No. 6/2009. (IV. 14) KvVM-EüM-FVM, 2009). Zinc, Pb, and Co contents were by far lower than the (B) limit value, as follows: Zn (64.3 vs 200 mg/kg), Pb (11.1 vs 100 mg/kg), and Co (8.8 vs 30 mg/kg). Although the Cu contents were lower than the pollution limit value (B) of 75 mg/kg, a clear Cu enrichment can be highlighted in the topsoil. Copper accumulation in the topsoil is an increasing problem in conventional and organic vineyards too. Indeed, the average Cu content (54.5 mg/kg) detected in the vineyard topsoil in Tokaj is comparable with that in vineyards within the 25 member states of the European Union (49.26 mg/kg) and noticeably higher compared to the overall average Cu concentration of 16.85 mg/kg in European soils (Ballabio et al., 2018).

Significant differences in Ni and Cr contents can be highlighted between the examined soil layers. Higher values in the subsoil (Fig. 2) at the 30–40 cm soil layer (Ni: 63.1 mg/kg and Cr: 125.5 mg/kg) suggest past accumulation. Increased Ni and Cr concentrations in specific subsoil layers exceed the pollution limit values in Hungarian standards (Ni: 40 mg/kg and Cr: 75 mg/kg) (Fig. 2). The spreading of organic fertilizers (for at least 27 years starting from the replanting of the vineyard in 1993) can be a plausible reason, as repeated applications of fresh manure, manure compost, and more recently, manure pellets may significantly increase Ni and Cr contents in cultivated soils (Barakat et al., 2016; Gong et al., 2019). Elevated concentrations of Cr and Ni observed at depths of 30–40 cm, where the plant roots are probably denser (according to Berlanas et al., 2019), may also pose a potential risk of toxicity, especially when replanting with young vine plants (Romić et al., 2004, Lago-Vila et al., 2015). However, those contents are still lower than the toxicity thresholds (200 mg/kg for Cr and 100 mg/kg for Ni) based on previous ecological risk assessment studies (MEF, 2007; Toth et al., 2016). Also, the high subsoil concentrations (at the soil depth of 30–40 cm) of Ni and Cr can be derived from the weathering of the dacite base rock (Grove et al., 2004) or the dust depositions during the extraction of the dacite rocks in the nearby Binét quarry. The quarry abandoned in the 1980s can be a plausible source of the neighboring vineyard's elevated Ni and Cr contents.

Correlation analysis revealed significant positive relationships between the Zn, Pb, Cu and the SOM contents (Table 2). The SOM content can play a dominant role in the vertical distribution of Zn, Cu and Pb. Higher contents of Cu, Zn, and Pb tend to prevail in soils rich in SOM compared to those with a poor SOM content (Rinklebe et al., 2019; Shaheen et al., 2017). The soil pH and the carbonate content are seemingly insignificant sources of variation for the soil-bound PTE contents in the studied vineyard. However, the lability of PTEs depends on the carbonate and the soil pH (Bradl, 2004) that are therefore important parameters in assessing PTEs. A significant positive relationship was observed between Cu and the Arany Plasticity Index and the silt percentage that confirms the affinity of Cu to fine-grained soil fractions, such as silt (Besnard et al., 2001; Brunetto et al., 2016; Fernández-Calviño et al., 2008, 2012; Rinklebe et al., 2019). In contrast, Zn and Cu showed negative correlation with the clay content. This negative relationship can be explained by the frequent fungicide and fertilizer applications which mainly increase the Cu and Zn contents in the soil surface, slightly depleted in its clay fraction compared to the subsoil (Brunetto et al., 2014). In some previous works, PTEs were also strongly associated with Fe-Al oxides (bounded and/or occluded) exhibiting a high surface area (Rinklebe et al., 2019; Scheinost, 2005). Indeed, the relationship between Ni and Cr contents and total Fe and Al in the vineyard soil in Tokaj indicates their association with Fe and Al oxides, as described in another forthcoming paper of our group (Pham et al., forthcoming paper, unpulished ).

**Table 2:** Spearman's correlation matrix comparing pH, soil organic matter (SOM), carbonate content, Arany Plasticity Index, and particle-size distribution with the examined PTE contents in all soil sampled layers, and separately in the topsoil (0–20 cm depth) and in the subsoil (> 20 cm depth)

	SOM	pH	CaCO_3_	Arany Plasticity Index	Clay	Silt	Sand
*All soil layers*							
Zn	0.95**	– 0.01	– 0.28	0.46	– 0.53*	0.24	0.30
Pb	0.64**	– 0.28	– 0.30	0.24	– 0.31	– 0.09	0.50*
Co	– 0.48	0.00	0.10	0.01	0.55*	0.01	– 0.50*
Ni	– 0.48	– 0.20	0.12	– 0.46	0.13	– 0.29	– 0.00
Cr	– 0.29	– 0.17	– 0.19	– 0.39	0.00	– 0.21	0.03
Cu	0.77**	– 0.07	– 0.05	0.48	– 0.51^*^	0.19	0.33
*Topsoil*							
Zn	0.78**	– 0.02	– 0.18	0.38	– 0.46	0.26	– 0.12
Pb	0.40	– 0.27	– 0.17	0.21	– 0.44	– 0.02	0.20
Co	– 0.01	0.38	0.40	0.60	0.04	0.65*	– 0.62
Ni	– 0.40	– 0.12	0.40	0.05	– 0.03	– 0.15	0.16
Cr	– 0.39	– 0.23	– 0.34	– 0.12	0.04	– 0.42	0.32
Cu	0.44	– 0.23	0.21	0.71*	– 0.48	0.27	– 0.13
*Subsoil*							
Zn	0.96**	– 0.63	– 0.64	0.07	0.00	– 0.29	0.43
Pb	0.94**	– 0.56	– 0.70	– 0.07	0.12	– 0.57	0.65
Co	0.29	– 0.62	– .835*	– 0.29	0.65	– 0.56	– 0.06
Ni	– 0.23	– 0.15	– 0.13	– 0.79*	– 0.06	– 0.27	.073
Cr	– 0.25	– 0.20	– 0.13	– 0.82*	– 0.02	– 0.31	0.09
Cu	0.86^*^	– 0.38	– 0.40	– 0.46	– 0.25	– 0.61	0.90**

*Significant at the level of *p* < 0.05

**Significant at the level of *p* < 0.01

Enrichment ratios were applied to assess PTE enrichment in eroded sediments compared to the vineyard topsoil (< 20 cm depth) (Fig. 2). Zinc (ER = 1.35), Pb (ER = 1.30), Co (ER = 1.32), Ni (ER = 1.27), and Cr (ER = 1.25) exhibited significantly higher contents in the sediments compared to the vineyard topsoil. The pseudo-total concentration of Cu ranging between 148.1 and 211.5 mg/kg was substantially greater than its topsoil levels (range: 36.5—70.0 mg/kg). Accordingly, the highest ER was also observed for Cu (3.36). Elevated concentrations of PTEs are often detected in sediments moved by rainfall-runoff in sloping vineyards (Devi et al., 2018; Komárek et al., 2010). The preferential association of PTEs with the organic-rich sediments (3.14 ± 0.23%) can explain their enrichment. Erosion and subsequent sedimentation of organic-rich suspended material and the post-deposition association of PTEs with organic-rich deposits within the low-energy zones of the terrain may be an important process driving the heterogeneous distribution of such elements in the topsoil (Rinklebe & Shaheen, 2014; Rinklebe et al., 2019). In addition, the eroded sediments are suggested to be enriched in silt-sized particles and depleted in coarser particles (Fernández-Calviño et al., 2008). Such fine-grained particles are generally considered an important contributor to the accumulation of PTEs in soils and sediments. The high contents of Cu in eroded sediments indicated that an appreciable part of anthropogenic Cu from treatments with organic amendments (fresh cattle manure, cattle manure pellets) and Cu-based fungicides might be moved and eventually exported off-site during rainfall-runoff events, as earlier observed in vineyard areas (Babcsányi et al., 2016; Besnard et al., 2001; Fernández-Calviño et al., 2008; Ribolzi et al., 2002). Therefore, a plausible risk of the translocation of PTE-polluted soil from vineyards into adjacent areas and surface water environments raises ecological risk concerns.

### Bioavailable contents of PTEs in the vineyard soil and sediments

**Fig. 3:**
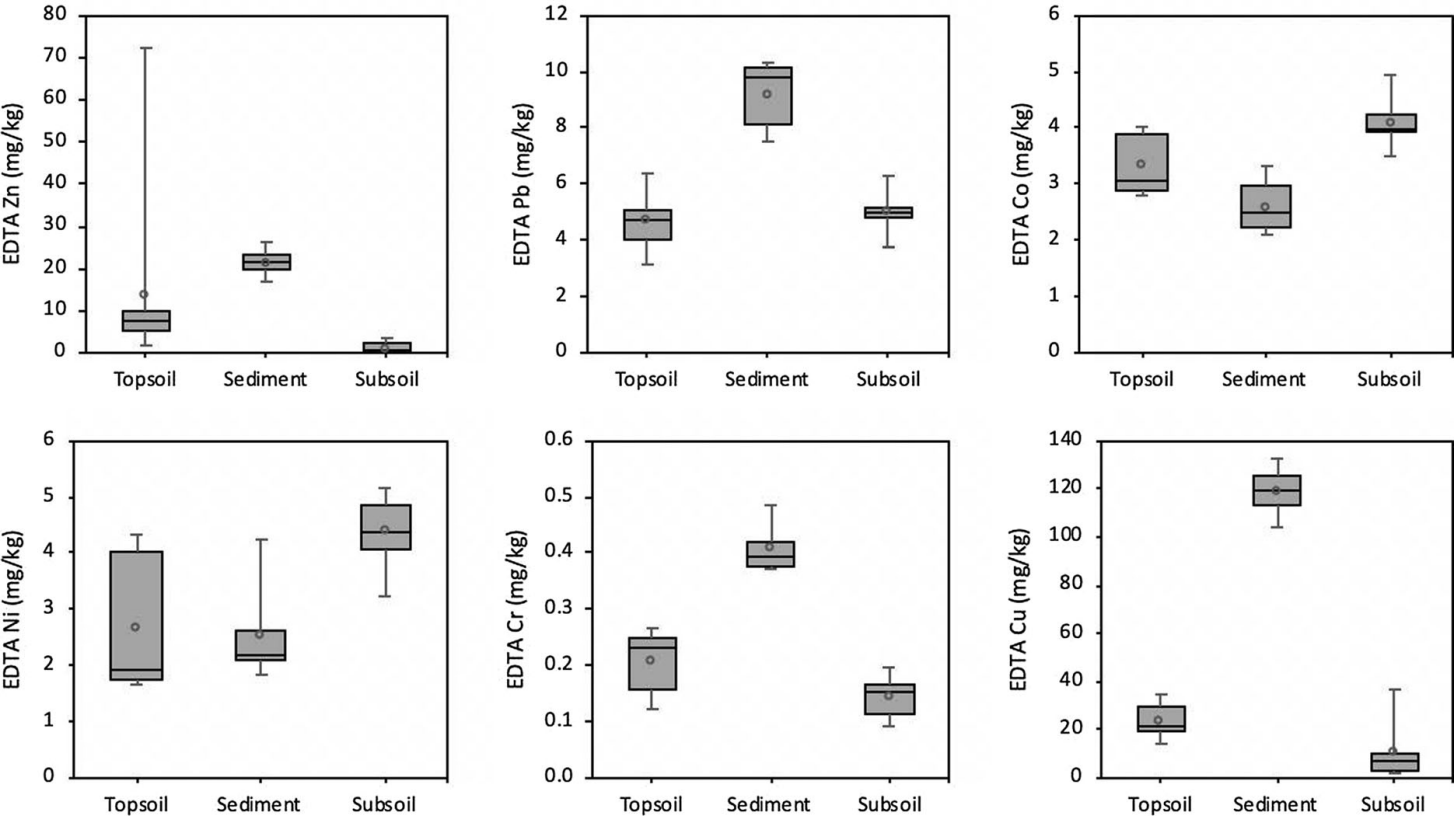
Bioavailable contents of the target PTEs (mg/kg) in the top- (0–20 cm), subsoil (> 20 cm), and the eroded sediments in the studied vineyard

The total PTE content is insufficient for an adequate environmental risk assessment because the mobility of these elements depends on their binding forms (Fernández-Calviño et al., 2012; Rinklebe & Shaheen, 2014; Szolnoki and Farsang 2013). Meanwhile, the bioavailability of PTEs has been used as a useful tool for evaluating the environmental pollution risk and toxicity to crops (Borgese et al., 2013; Nunes et al., 2014; Violante et al., 2010). Typically, the EDTA extracted PTE contents (marked with X_E_) in the sediments were higher than those in the vineyard top- and subsoil, except for Co and Ni (Fig. 3). Copper showed substantial lability in the eroded sediments compared to the vineyard soils (Fig. 3). In addition, the important proportions of bioavailable Cu in the topsoil, sediment, and subsoil (with bioavailable ratios of 48%, 63%, and 37%, respectively) indicated its weak binding (Table 3). At the same time, EDTA salt was formerly found to overestimate Cu_E_ content, especially in eroded sediments with a high organic matter content, due to its capability of dissolving high molecular weight organic compounds and of sequestering the Cu from the organic matter binding sites (McBride et al., 1998). Similarly, high bioavailability ratios of Pb and Co in the vineyard soil and sediment indicated their lability, particularly in the sediments (66%) and subsoil (53%) for Pb, and in the top- and subsoil for Co (33 and 45%) (Table 3). Therefore, assessment of Pb-associated ecological risk is also required for vineyards (especially for children) (Rinklebe et al,. 2019; Tirima et al., 2016). Cobalt has received so far little attention in assessing PTE-associated ecological risks, yet Co is broadly considered as an element in the high-risk category (Barrio-Parra et al., 2017). Median EDTA-extracted contents of Co and Ni in the subsoil were higher than those in the sediment (Co: 4.0 vs 2.5 mg/kg and Ni: 4.5 vs 2.2 mg/kg), indicating the mobilization of particles bearing less bioavailable Ni and Co. The Cr_E_ content in the sediments was markedly higher than that in the top- and subsoil, which contrasted its lower total content in the sediment. Overall, bioavailability ratios of Ni and Cr in both vineyard soil and sediments were low, accounting for 9% and 1%, respectively (Table 3).

**Table 3:** Median of bioavailability ratio* (%) of target PTEs in the soil and sediments

	Zn	Pb	Co	Ni	Cr	Cu
Topsoil	13	39	33	6	1	48
Sediment	22	66	21	6	1	63
Subsoil	7	53	45	9	0	37

*Bioavailable Ratio (%) = (Bioavailable metal fraction/pseudo-total metal concentration in sample material) × 100 (Alibrahim & Williams, 2016; Kashem & Singh, 2001)]

The Zn_E_ contents in the topsoil showed a great range of variation (range 1.9–72.3 mg/kg) and represented on average 17% of the pseudo-total Zn. Bioavailable fractions of Zn were either not detected or negligible in the subsoil. A higher median Zn_E_ content was found in the sediment (21.6 mg/kg) compared to the vineyard topsoil (7.3 mg/kg), the former accounting for 22% of the total Zn contents (Table 3). Zinc was reported as an element presenting low mobility in alkaline soils, mainly bound to and incorporated into minerals (Antoniadis et al., 2017; Brunetto et al., 2014, 2016; Duplay et al., 2014). Likewise, Zn in eroded sediments showed similar geochemical partitioning mainly detected in the residual and organically bound fractions (Canuto et al., 2013; Fernández-Calviño et al., 2012).

Similarly to the total PTE contents, the soil pH was not a significant source of variation for their bioavailable proportion in the soil. On the other hand, the SOM content strongly influenced the EDTA extracted contents of target PTEs (Table 4). A positive correlation with the SOM content for Zn_E_ and Cu_E_ may be driven by the soil pH of the studied vineyard soils. Indeed, the alkaline pH in former researches promoted organic matter solubilization and hence the release of complexed Cu from soils (Brunetto et al., 2016; Fernández-Calviño et al., 2008), while Zn release presumably occurred through its release from Al and Fe oxyhydroxides (Fernández-Calviño et al., 2012). On the other hand, the bioavailable contents of Co and Ni were negatively correlated with the SOM in soils in line with their lower EDTA-extracted contents in the runoff-transported sediments. Overall, the low mobility of Co and Ni in the studied alkaline soils and a lack of SOM-related enrichment in sediments during erosive rainfall events further corroborate their geogenic origin.

**Table 4:** Spearman's correlation matrix of pH, soil organic matter (SOM), carbonate content, Arany Plasticity Index, and particle-size distribution compared with the EDTA extracted PTE contents (marked with X_E_) in all soil layers, and separately in the topsoil (0–20 cm depth) and in the subsoil (> 20 cm depth)

	SOM	pH	CaCO_3_	Arany Plasticity Index	Clay	Silt	Sand
*All soil layers*							
Zn_E_	0.84**	– 0.39	– 0.29	0.24	– 0.49*	0.01	0.41
Pb_E_	– 0.13	– 0.28	– 0.09	– 0.18	– 0.20	– 0.78**	0.78**
Co_E_	– 0.66**	– 0.09	– 0.09	– 0.45*	0.44	– 0.46*	0.11
Ni_E_	– 0.55*	– 0.14	– 0.21	– 0.35	0.42	– 0.62**	0.26
Cr_E_	0.53*	– 0.11	0.19	0.36	0.05	0.25	– 0.24
Cu_E_	0.78**	– 0.34	– 0.12	0.48^*^	– 0.54*	– 0.01	0.40
*Topsoil*							
Zn_E_	0.77**	– 0.61	– 0.52	0.27	– 0.42	0.14	0.07
Pb_E_	– 0.38	– 0.46	– 0.10	– 0.03	– 0.22	– 0.79**	0.83**
Co_E_	– 0.72*	– 0.01	0.29	– 0.10	0.01	– 0.64*	0.67*
Ni_E_	– 0.71*	0.02	0.28	– 0.25	0.15	– 0.70*	0.67*
Cr_E_	0.72*	– 0.17	0.03	0.46	0.15	0.67*	– 0.58
Cu_E_	0.56	– 0.16	0.06	0.52	– 0.41	0.60	– 0.31
*Subsoil*							
Zn_E_	0.64*	– 0.35	– 0.27	– 0.27	– 0.30	– 0.81**	0.77**
Pb_E_	0.58	– 0.28	– 0.20	– 0.18	– 0.44	– 0.74*	0.78**
Co_E_	– 0.03	– 0.36	– 0.60	– 0.70*	0.42	– 0.04	– 0.14
Ni_E_	0.26	– 0.57	– 0.85**	– 0.38	0.34	– 0.33	0.20
Cr_E_	0.28	0.07	0.39	0.00	0.14	– 0.36	0.00
Cu_E_	0.78**	– 0.42	– 0.44	0.03	– 0.23	– 0.87**	0.82**

*Significant at the level of *p* < 0.05

**Significant at the level of *p* < 0.01

### Soil contamination indices

**Fig. 4:**
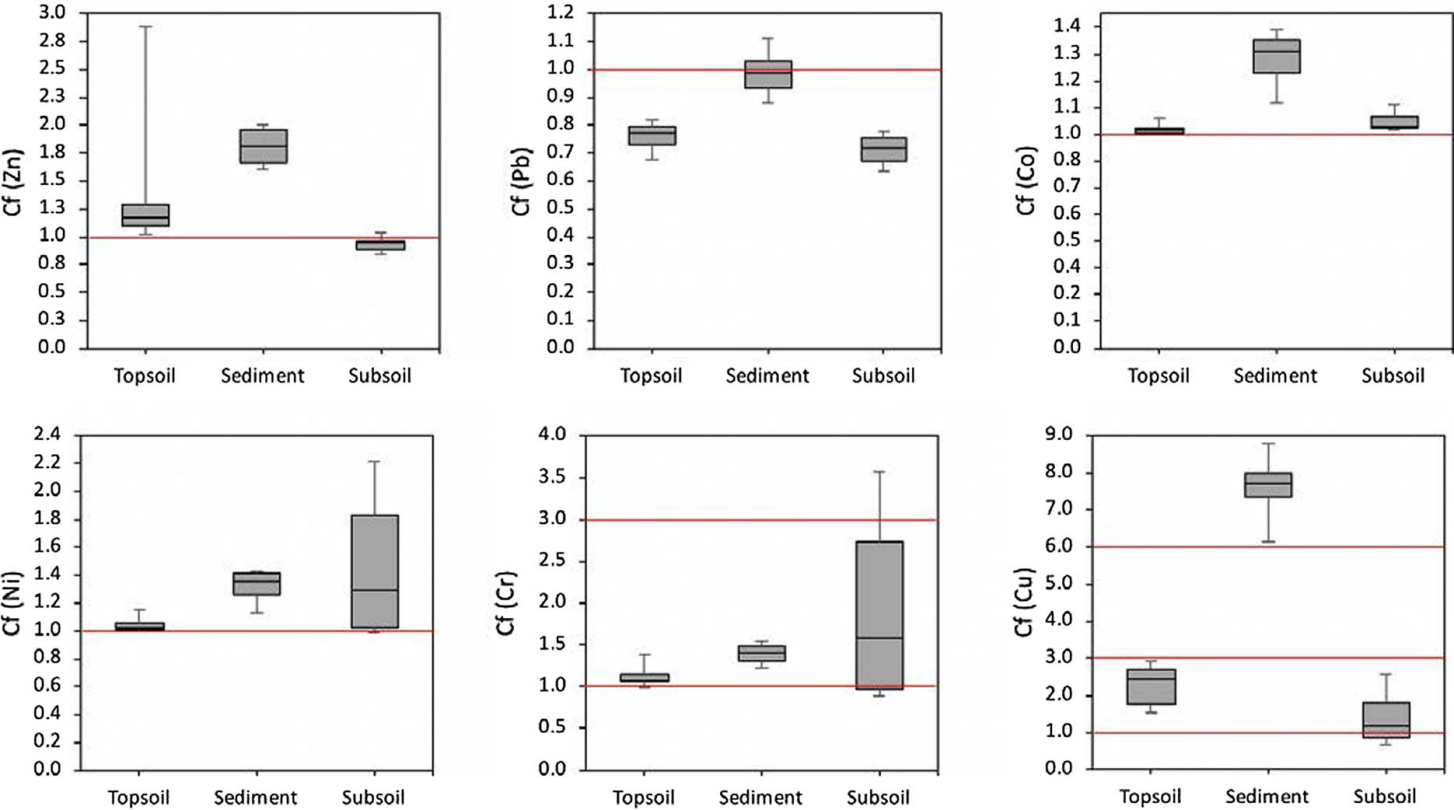
Contamination factor (Cf) of PTEs in the top- (0–20 cm) and subsoil (> 20 cm) and sediments. The red lines highlight Cf = 1, above which contamination is identified; Cf = 3, above which considerable contamination is identified; Cf = 6, above which high contamination is identified

The PTE-based contamination factors (Cfs) in the sediment were higher than those in the vineyard soil, except for Ni and Cr (Fig. 4). The median Cfs in the sediments for Zn (1.8), Co (1.3), Ni (1.4), and Cr (1.4) imply a moderate contamination level. The high Cfs for Cu (ranging from 6.2 to 8.8) indicate significant contamination and strong enrichment of Cu compared to the local forest soil. In general, moderate contamination was observed for all examined PTEs in the vineyard top- and subsoil, except for Pb.

**Fig. 5:**
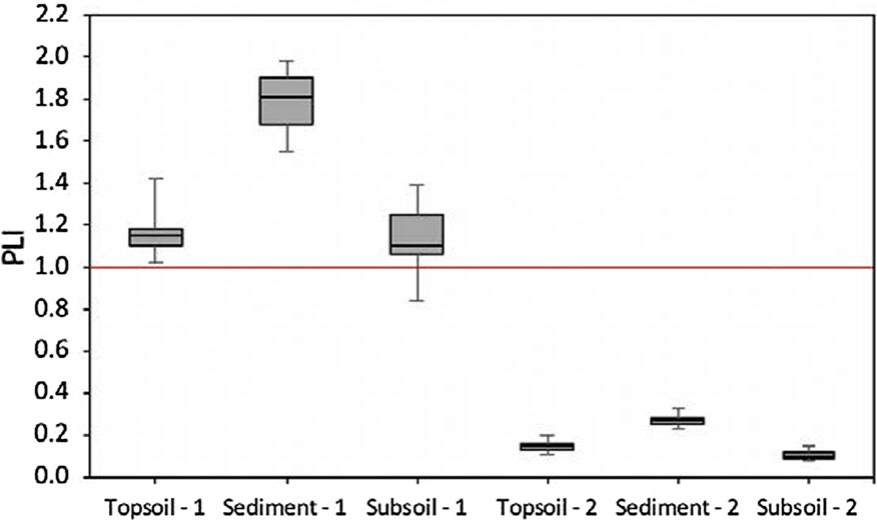
The pollution load index (PLI) of the top- (0–20 cm), subsoil (> 20 cm), and sediments in the studied vineyard (1–calculated with the total PTEs; 2–calculated with EDTA extracted PTEs). The red line displays PLI = 1, above which pollution is identified

The calculated contamination indices showed that Cu, Zn, Ni, and Cr predominantly accumulated in the studied vineyard soils and were significantly enriched in the eroded sediments, causing an elevated pollution risk, in particular, for Cu. The pollution load index (PLI) encompassing all individual Cf values varied from 1.01 to 1.42 and from 0.84 to 1.39 for vineyard top- and subsoils, respectively, classifying the vineyard soils in no to moderate pollution status. Meanwhile, the higher PLI for the eroded sediments, ranging from 1.55 to 1.98 (Fig. 5), belongs to the moderate pollution category. Significant contamination is detected when the PLI value exceeds 1 (Rinklebe et al., 2019), demonstrating that the studied vineyard soils and sediments represent a multi-element contamination case.

### Ecological risk assessment

Former studies identified the ecological risk index (ERI) as one of the most powerful  indices for assessing the overall ecological risk in soils and sediments (Arfaeinia et al., 2019; Luo et al., 2007; Liu et al., 2014). Based on the ecological risk indices (Ei) summarized in Table 5, the soil and eroded sediments represent a low ecological risk. Indeed, all calculated values are below the ecological risk thresholds of 40 for Ei and 90 for ERI. The ecological risk indices showed no elevated ecological risk in the studied organic vineyard; meanwhile, Mirzaei and co-workers (2019) reported the range of ERI (for Cu, Zn, Pb, Cr, and Cd) in 38 long-term fertilized vineyards from 5.30 to 468.09, indicating considerable ecological risk for 6 vineyards representing a moderate risk and 3 vineyards a high risk. Considering bioavailable PTE contents for the ecological risk assessment (Table 5), which depict an even more realistic estimation of environmental threats, the calculated indices are lower and show a negligible risk.

**Table 5:** The average values of the ecological risk indices for target PTEs in the top- (0–20 cm) and subsoil (> 20 cm) and eroded sediments (EDTA extracted PTE contents are marked with X_E_). Ei stands for the ecological risk factor, while ERI is the ecological risk index

	Ei (Topsoil)	Ei (Sediment)	Ei (Subsoil)
*Pseudo-total metals*			
Zn	1.3	1.8	0.9
Pb	3.8	4.9	3.6
Co	4.9	6.5	5.1
Ni	6.2	7.9	8.7
Cr	2.2	2.8	3.8
Cu	11.3	38.0	7.0
ERI	29.8	62.0	29.2
*EDTA extracted metals*			
Zn_E_	0.2	0.4	0.0
Pb_E_	1.6	3.2	1.7
Co_E_	1.9	1.4	2.3
Ni_E_	0.6	0.5	0.9
Cr_E_	0.0	0.0	0.0
Cu_E_	4.9	24.6	2.2
ERI_E_	9.2	30.2	7.1

As expected from the Ei data for the target PTEs, the two highest contributors to the ERI were Cu (Ei (average): 11.3 in the topsoil, 38.0 in the sediment, and 7.0 in the subsoil), followed by Ni (6.2 in the topsoil, 7.9 in the sediment, and 8.7 in the subsoil). The relative contribution to the overall ecological risk of the other examined PTEs was negligible. The highest values of Ei were assessed for Cu, especially in eroded sediments (Ei (pseudo-total Cu) = 38.0, accounting for 61.3% of the total ecological risk). The disparity was noticeable for the bioavailable fraction of Cu in the vineyard soil and sediment. The figures for bioavailable Cu contribution to the ERI in eroded sediments reached its maximum Ei of 24.6 and made up 82% of the total ecological risk. The latter revealed the prevailing influence of bioavailable Cu in the overall ecological risk represented by the six target PTEs in the vineyard. In addition, the most noticeable difference between the distribution percentage of pseudo-total and bioavailable Cu relative to the total ERI indicated an apparent increase in the ecological risk prevailing in the sediments, especially for the EDTA extracted fractions.

## Conclusions

Cultivation in steep areas such as sloping vineyards exerts a significant impact on the soil environment and affects the spatial distribution of potentially toxic elements. Soil erosion and the long-term use of chemical fertilizers, fungicides, and organic byproducts can raise the pollution and ecological risk related to potentially toxic elements (PTEs) in vineyards. The soil and eroded sediments demonstrated a multi-element contamination case in the organic vineyard with PLI exceeding 1. Significant enrichment was apparent for pseudo-total contents of Cu in the topsoil attributed to Cu-based fungicides' use. Differently, Ni and Cr showed a noticeable accumulation in the subsoil (originated predominantly from geogenic sources or manure applications), displaying a moderate contamination level. Consequently, a potential risk of toxicity may arise, especially in the rhizosphere area.

On the other hand, the target PTEs tented to enrich significantly in the eroded sediments. Copper exhibited higher contents in the sediments (148.1–211.5 mg/kg) compared to those in the vineyard topsoil (range: 36.5–70.0 mg/kg). Accordingly, the highest ER was also determined for Cu (ER > 3), revealing its marked enrichment. Contamination factors also showed significant Cu pollution compared to the reference neighboring forest soil. Despite high pseudo-total Cu contents in the soil, the pollution threshold was not exceeded according to Hungarian national standards. A moderate enrichment of Zn was also observed in the vineyard topsoil compared to the subsoil, likely due to inputs through micronutrient fertilizer applications.

The examined PTEs demonstrated higher bioavailable contents in the eroded sediments than the top- and subsoil, except Ni and Cr. The predominant bioavailable nature of Cu in the sediment samples (63% of pseudo-total Cu) revealed a plausible ecological risk. Although high bioavailability ratios of Pb and Co in the soil and sediments indicated their lability in the vineyard, soil pollution by Pb and Co was not evidenced. Zinc was moderately bioavailable, while Ni and Cr were immobile with bioavailable ratios less than 9% and 1%, respectively.

Ecological risk assessment based on the pseudo-total and bioavailable contents of PTEs showed a low risk (ERI < 90) in the studied vineyard. Copper was the dominating risk factor regardless of its forms (total and bioavailability) in the entire ecological risk. The high bioavailable Cu contents in eroded sediments accounted for up to 82% of the total ecological risk by all examined PTEs. The latter revealed an apparent risk induced by the high lability of Cu in the runoff-transported sediments, which is expected to increase over time with the repeated use of Cu-based agrochemicals.
